# "No touch" technique and hypothermic circulatory arrest for porcelain aorta in combined valve surgery

**DOI:** 10.1186/1749-8090-10-S1-A173

**Published:** 2015-12-16

**Authors:** R Leyh, C Bening, C Schimmer, M Oezkur, A Gorski, K Hamouda

**Affiliations:** 1 University of Wuerzburg, Dept. of Thoracic and Cardiovascular Surgery, Oberduerbacher Str, 6 97080 Wuerzburg, Germany

## Background/Introduction

Cardiac surgery in patients scheduled for combined single or multiple valve and CABG surgery with preoperatively undetected porcelain aorta is challenging. Different surgical strategies may address this problem. Abortion of the initial operation with subsequent interventional therapy and hypothermic circulatory arrest offer clampless treatment options in these patients.

## Aims/Objectives

The aim of this retrospective study is to characterize patients with preoperatively undetected porcelain aorta scheduled for combined single or multiple valve and CABG surgery that were treated with hypothermic circulatory arrest during operation addressing the porcelain aorta and to describe the outcome.

## Method

From 01/2011 to 04/2015, 19 patients (74.8 ± 7.4years, 39% female) with preoperatively undetected porcelain aorta and combined single or multiple valve and CABG surgery were observed. 15 patients (79%) presents with aortic valve pathology and CAD and 5 patients (26%) with aortic valve pathology ± CAD and mitral or tricuspid valve pathologies. In all patients the ascending aorta ± hemiarch was replaced using circulatory arrest. The Euro Score II was 12.7 ± 4.4%.

## Results

Mean cardiopulmonary bypass, cross clamp and hypothermic circulatory arrest times were, respectively, 140 ± 48 minutes, 96 ± 37 minutes and 11.9 ± 3.3 minutes. Bladder and tympanic temperature were, respectively, 27.9 ± 2.3°C and 23.3 ± 2.8°C. The 30 day mortality was 10.5% (n = 2), Stroke occurred in 5.3% (n = 1), renal failure in 15.8% (n = 3), prolonged ventilation was necessary in 21% (n = 4) and 10.5% (n = 2) had to be reoperated for bleeding, mean ICU stay was 4.4 ± 2.4 days. The mean length of hospital stay was 11.5 ± 4.4 days.

## Discussion/Conclusion

These preliminary data indicates that hypothermic circulatory arrest with a "no touch" technique is a reasonable safe and reproducible surgical option in patients with preoperatively undetected porcelain aorta scheduled for combined single or multiple valve and CABG surgery. However, better preoperative diagnosis is necessary to select patients for different treatment strategies, surgery vs. interventional therapy and to conduct larger studies comparing the results of different treatment strategies in these high risk patients.

